# The prevalence of osteoarthritis: Higher risk after transfemoral amputation?—A database analysis with 1,569 amputees and matched controls

**DOI:** 10.1371/journal.pone.0210868

**Published:** 2019-01-22

**Authors:** Bastian Welke, Eike Jakubowitz, Frank Seehaus, Kiriakos Daniilidis, Matthias Timpner, Nils Tremer, Christof Hurschler, Michael Schwarze

**Affiliations:** 1 Laboratory for Biomechanics and Biomaterials, Department of Orthopaedic Surgery, Hannover Medical School, Hannover, Germany; 2 OTC, Orthopädie Traumatologie Centrum Regensburg, Regensburg, Germany; 3 Techniker Krankenkasse, Hauptverwaltung - Fachbereich GPH, Hamburg, Germany; Holland Bloorview Kids Rehabilitation Hospital, CANADA

## Abstract

**Background:**

Several studies have shown that patients with a unilateral amputation have an increased risk of developing osteoarthritis (OA) in the knee of their sound leg. OBJECTIVE: The first objective was to investigate whether amputees are more frequently affected by gon-, cox- or polyarthritis as well as back pain or spinal disorders. We hypothesized that mobile and active transfemoral amputees more often experience OA and spinal disorders than non-amputees. The second objective was to compare the mean age of the patients with OA.

**Patients:**

Patients with a unilateral transfemoral amputation (n = 1,569) and five abled-body control groups (each n = 1,569) matched in terms of age and gender resulting in total of 9,414 participants.

**Methods:**

Groups were analyzed regarding the prevalence of six selected diagnoses regarding musculoskeletal disorders.

**Results:**

A significantly decreased prevalence of OA and specific disorders of the spine in transfemoral amputees compared to a control group was found. The amputees with OA are significantly younger than patients with OA in the control group.

**Conclusion:**

The results from the presented study contradict previously published literature. Apparently circumstances of life play an important role, like physical work and strenuous activities which are likely to be underrepresented in the amputee group. The results of the study need to be used cautiously due to the major limitation of the study which is the lack of detail in individual patients caused by the methodology.

## Introduction

The primary goal of amputation of extremities is the removal of life-threatening tissue related to e.g. trauma, malignancy, vascular and neural conditions and the creation of a preferably peripheral and mechanically strong residual limb. The most common cause of lower extremity amputation in industrialized countries is chronic vascular disease in nearly 70% of cases, followed by trauma with over 20% [1]. Worldwide, diabetes is the most frequent cause of lower extremity amputation [2]. The number of annual major amputations of the lower extremity in Germany has steadily increased from about 20,000 in the 1960s [3] to nearly 61,000 in 2003 [4]. In 2005 over 1 million citizens of the United States were live with the loss of a limb after an amputation of the lower extremity [5].

Studies have shown that patients with a unilateral amputation have an increased risk of developing osteoarthritis (OA) in the knee of their sound leg [6–10]. Norvell et al. reported a prevalence of knee OA of 11.7% for non-amputees compared to 16.1% for amputees [6]. Additionally, the proportion of people experiencing knee pain was more than two times higher for the amputees. OA is more prevalent in the sound limb of amputees [11]. Amputees with a higher mobility grade tend to load their sound limb more extensively during activities of daily living as well as to relieve the residual side. Several authors have speculated that this asymmetry in gait patterns and an increased load on the sound limb could lead to a higher incidence of OA [7,12]. On the other hand, less than 30% of transfemoral amputees achieve independent mobility outside of the home environment [13]. This reduced activity likely decrease the wear of joints and therefore may reduce the incidence of OA. In the present study, the risk of OA of amputees with an above average mobility was investigated.

The first objective was therefore to investigate whether mobile amputees are more frequently affected by OA or, as a result from asymmetric loading, back pain and spinal disorders. We hypothesized that mobile and active transfemoral amputees more often experience OA than non-amputees. The second objective was to compare the mean age of amputees and controls with OA, since there is no data available in literature. In order to achieve a high number of patients, a database analysis in collaboration with a German health insurance company was carried out.

## Methods

The database of the largest health insurer in Germany (Techniker Krankenkasse (TK)), which has about 9.5 million insured members, was analyzed in a five year interval (2010–2014, accessed and analyzed in January 2015). All data were fully anonymized by the insurer before they were accessed by the authors. Therefore, no IRB approval had to be obtained from the local ethics committee. In order to include amputees with a high mobility grade (MFCL classification: K-Level 3 and 4: at least community ambulator) within this database search, an inclusion criteria was that the amount of a single position in the intervention costs was higher than 20,000 EUR [14,15]. This cost is the lower threshold for a treatment with a micro-processor controlled knee prosthesis, which are usually worn by mobile patients. Additionally, patient files responding to a search term with common microprocessor-controlled knee prosthesis types were included. Patients were included irregardless of whether it was an initial treatment, a subsequent treatment or repair/service of the prosthesis. For further analysis, patients were also taken into account if they had switched the insurance provider, left the TK or dropped out caused by death. This selection resulted in 1,569 patients with an amputation. Due to our inclusion criteria, the amputee cohort consists of transfemoral as well as out of also knee disarticulation all with micro-processor controlled knee prosthesis. Because of the rare prevalence of knee disarticulation in Germany with approximately 5% of all amputations of the lower extremity we decided not to differ between amputation levels for convenience [16].

**Fig 1:**
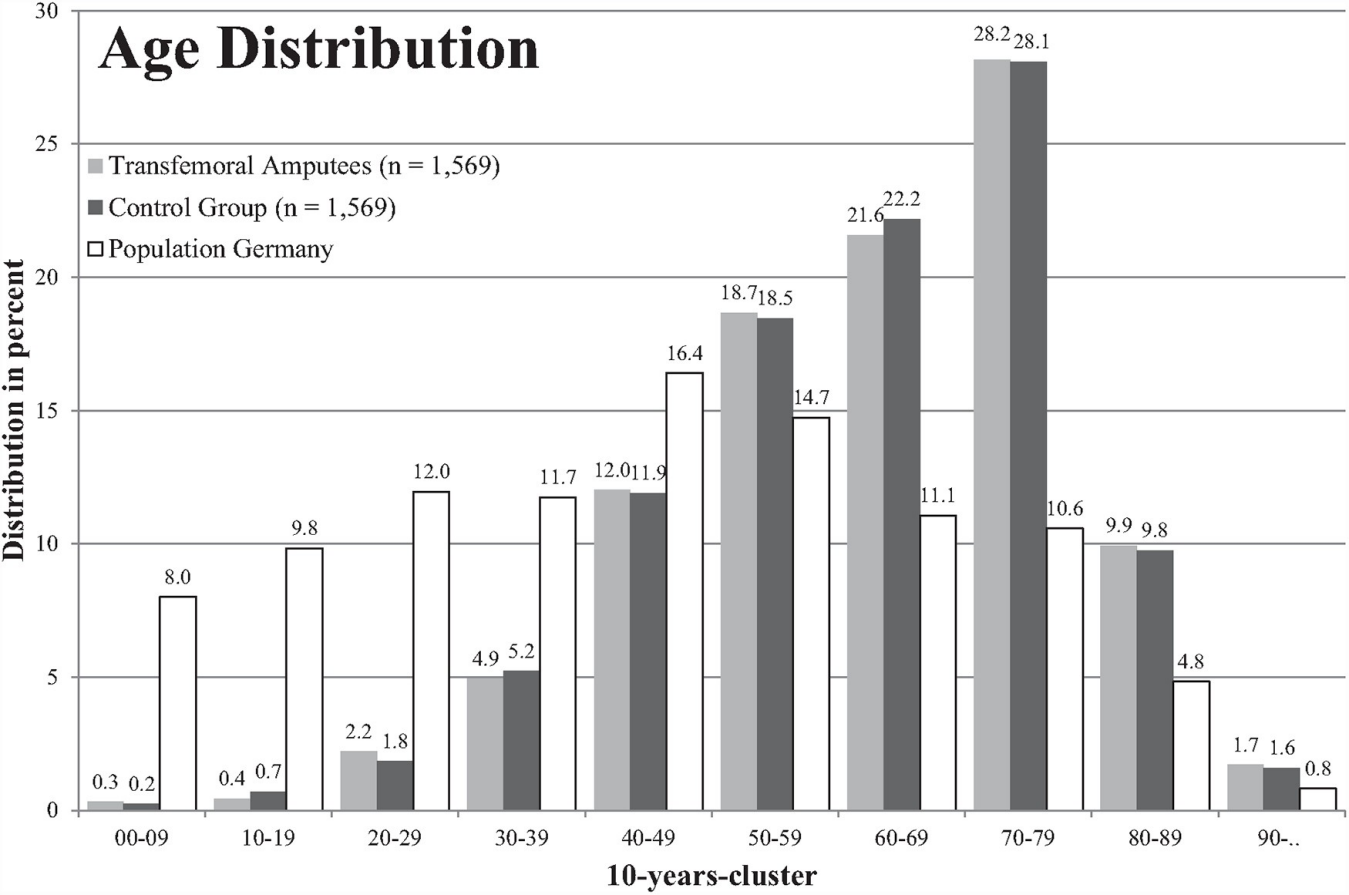
Age distribution of the amputee (light grey) and control group (black) compared to the general German population (blank).

For comparison purposes, five control groups without amputation were created from the same database. Each consisted of 1,569 patients and was matched in terms of age, gender, and drug expenses (Fig 1, S1 Table). The mean age of all groups was 63.3 years and is therefore around 20 years older than the median age of the German population which is approximately 44.5 years [17] (Fig 1, S2 Table).

### Data and statistical analysis

Groups were analyzed regarding the prevalence of six selected diagnoses regarding musculoskeletal disorders based on the 10^th^ revision of the International Classification of Diseases (ICD)[18]:

- M15—Polyarthrosis—arthrosis with mention of more than one side
- M16—Coxarthrosis—arthrosis of the hip
- M17—Gonarthrosis—arthrosis of the knee
- M42—Spinal osteochondrosis
- M43—Other deformities of the spine
- M54 –Back pain

The arthrosis diagnoses M15 to M17 and the spinal disorders M42 and M43 are assured by radiography by the diagnosing practitioner as required by standards. Multiple diagnoses occurring in one patient were considered as one disorder. Therefore, it could be determined how many insured have received a specific diagnosis in the observed period between 2010 and 2014.

The statistical analysis of the first objective, that mobile and active transfemoral amputees are more often affected by musculoskeletal disorders than non-amputees, was carried out using the Chi-squared test (S4 Table). The numbers of affected patients in the control group was calculated as the mean from the five randomly built groups. A significance level of alpha = .05 was applied. The second objective of the study was to clarify whether the insured affected by musculoskeletal disorders with a transfemoral amputation are younger than those in the control group (S3 Table). The mean age of the amputees was considered significantly lower if the upper limit of the 95% confidence interval (CI) of this group and the lower limit of the 95% CI of three of five control groups do not intersect.

## Results

Due to the selection methodology regarding, the age structure of the transfemoral amputees and the control group were very similar (Fig 1). For all six selected musculoskeletal disorders the number of insured in the control group was higher than in the group of the transfemoral amputees (Table 1). The differences between the two tested groups were significant with the exception of the diagnosis of gonarthrosis (M17). The hypothesis of the study was therefore rejected.

**Table 1:** Number of insured affected by the selected musculoskeletal disorders. Asterisks (*) denote values significantly different between the two groups (alpha = 0.05).

ICD-10	amputees group		control group		p-value
	number	percentage	number	percentage	
M15 Polyarthrosis	74	4.7%	111	7.1%	* p = .006
M16 Hip OA	176	11.2%	214	13.6%	* p = .045
M17 Knee OA	261	16.6%	302	19.2%	p = .063
M42 Spinal osteochondrosis	123	7.8%	194	12.4%	* p≤.001
M43 Other deformities of the spine	61	3.9%	91	5.8%	* p = .016
M54 Back pain	673	42.9	779	49.6	* p≤.001

**Fig 2:**
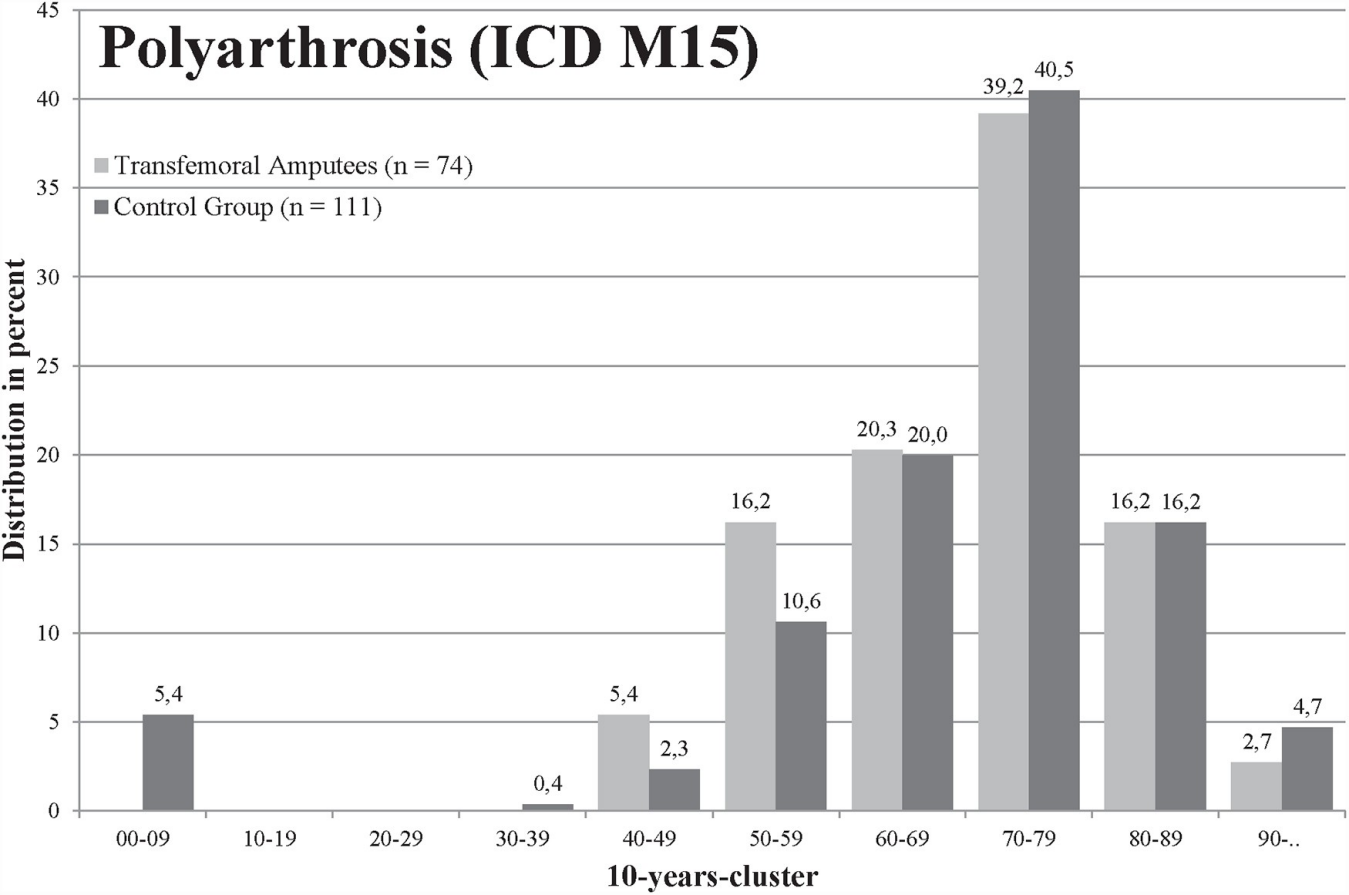
Age distribution of patients diagnosed with polyarthrosis (ICD M15). Amputee (light grey) and control group (black).

**Fig 3:**
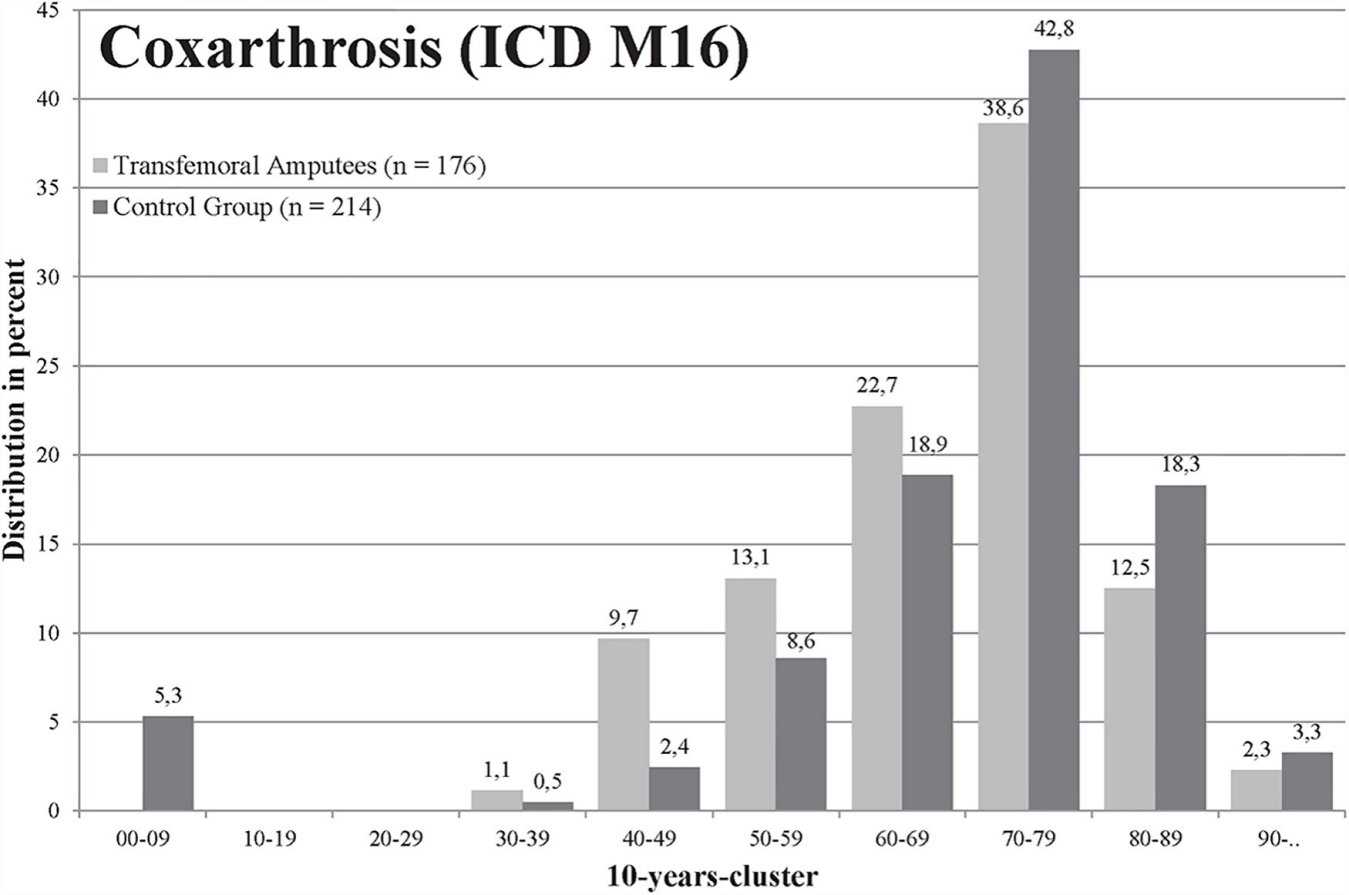
Age distribution of patients diagnosed with OA of the hip (ICD M16) of the amputee (light grey) and control group (black).

**Fig 4:**
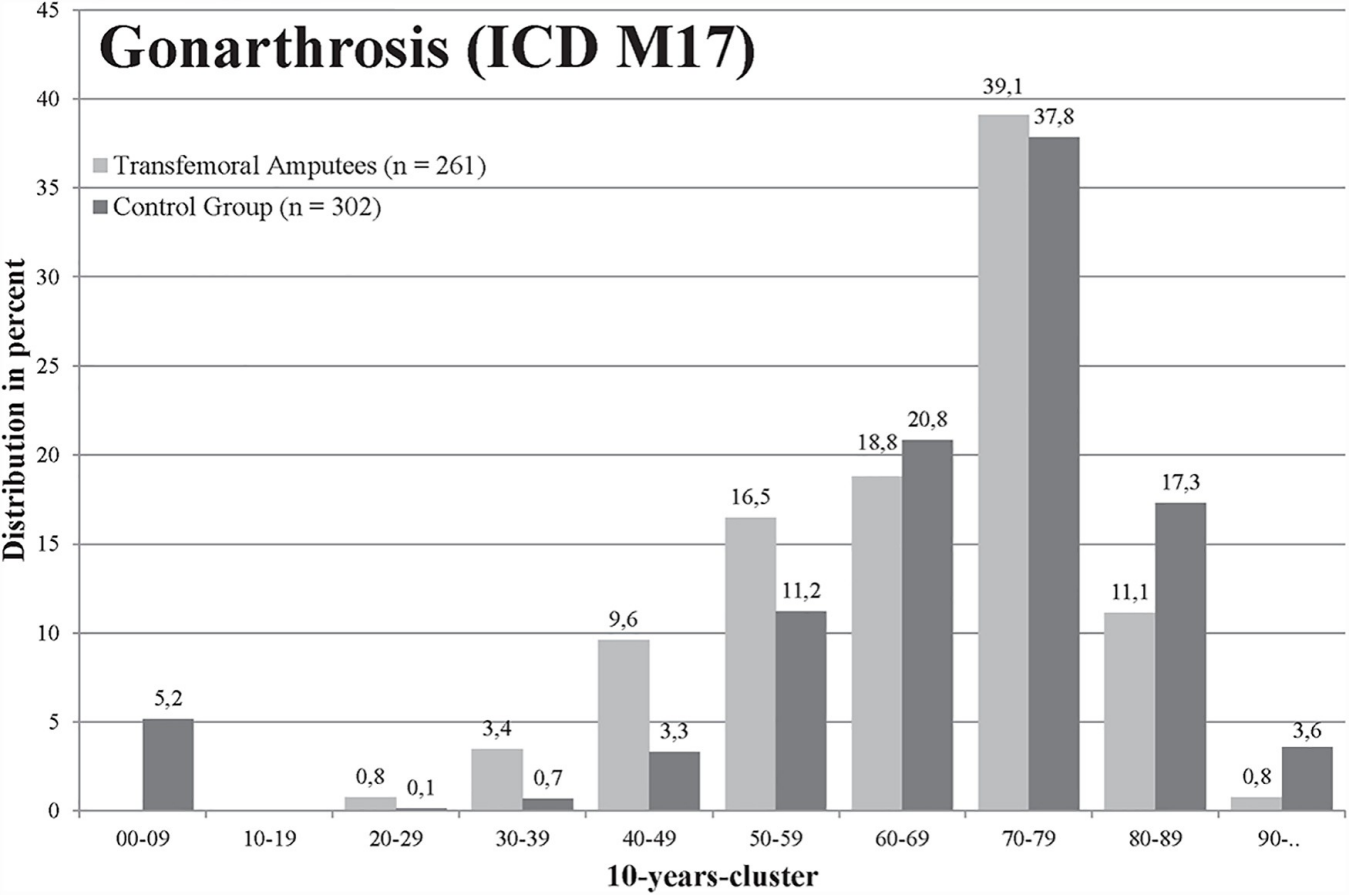
Age distribution of patients diagnosed with OA of the knee (ICD M17) of the amputee (light grey) and control group (black).

For polyarthrosis (M15), 4.72% insured in the group of transfemoral amputees and 7.07% insured in the control group were affected (p = .006, Table 1). The amputees were younger when they were affected by polyarthrosis, but there was no significant difference (mean age 69.5 to 71.9 years, Fig 2). A total of 11.22% amputees and 13.64% persons in the control group were affected by coxarthrosis (M16) (p = .045, Table 1), whereas amputees were significantly younger (67.9 to 72.3 years in the control group, Fig 3). Both groups were most frequently affected by gonarthrosis (M17), in total 563 of 3,138 (17.94%) insured, but the difference between groups was not significant (Table 1). The mean age of the amputees with gonarthrosis was significantly lower with 65.7 years compared to the control group with 71.1 years (Fig 4). For the diagnosis spinal osteochondrosis (M42) the amputees were significantly less frequently affected with 7.84% compared to 12.36% insured (p≤.001, Table 1). The lowest numbers of insured were affected from other deformities of the spine (M43). In the transfemoral amputee group, 3.89% persons were affected by other spine deformities and 5.80% insured in the control group (significant: p = .016, Table 1). The amputee group was significantly less affected by back pain (M54, p≤.001, Table 1).

## Discussion

For the first time the present study reports the prevalence of OA in patients with a transfemoral amputation supported by a large number of patients from a large database. Compared to matched controls, we found a significantly lower prevalence in five of six selected musculoskeletal disorders for amputees. Although the prevalence is lower, amputees are affected by OA at the hip and knee joint at a significantly younger age.

The results from the presented study contradict a couple of previous reports. For example Norvell et al. found a 1.5 fold increased prevalence of knee OA in amputees compared to non-amputees [6]. However, their numbers are based on a relatively small sample (amputees: n = 62, non-amputees: n = 94) and may, therefore, be associated with a high uncertainty (CI of prevalence factor: 0.6 to 3.4). In an observational study, Struyf et al. investigated 78 cases of traumatic leg amputees and their prevalence of OA [8]. They found a prevalence of 14% hip and 27% knee OA, whereas an age adjusted general population showed prevalence of 1.1% and 1.5% respectively. While the values for amputees are comparable to our results, the values of the general population are in high discrepancy to the values of our control group. This may be explained by the age structure of our controls since it is skewed towards the elderly and is about 20y older than the average population in Germany [17]. This in turn was caused by matching the age to the amputee group, where diabetes, which is associated with age, caused the majority of amputations [2,19]. Additionally, it is unclear how vascular insufficiency affects the onset or progress of OA in the contralateral knee joint of the amputees with vascular disorders.

Also Kulkarni et al. reported a three to sixfold higher prevalence of OA in amputees compared to an age matched population[9]. The mean age of the participants was with 73 years comparable to our groups. Nevertheless, they studied a specific population of Second World War veterans (n = 44) and since their amputation was exclusively caused by trauma, it seems not to be representative regarding average amputees with vascular disorders as the most relevant cause for amputation.

In a gait analysis study, Lemaire and Fisher concluded that increased loads within the amputee’s gait support the concept of increased knee OA risk of the sound limb [10]. Other gait analysis studies came to the same conclusions [12]. Within our study knee OA did not occur significantly more often, while all other disorders were significantly more present in the healthy control group. This is even more interesting, since our amputee group was solely composed of transfemoral amputees, which only possess one knee and therefore have a reduced incidence of knee OA.

Comparing our percentage of amputees experiencing back pain to previously published results from surveys, we found our numbers slightly lower with 42.9% than those results ranging from 52% to 87% [20–22]. These differences might partly be explained with our results based on diagnosed back pain and survey studies on self-reported back pain. This can also be observed in our reported prevalence of back pain in the control group with 49.6%, which is in the lower third of studies reporting lifetime prevalence of lower back pain[23]. Specifically for the German population, a life-time prevalence of back pain of 74% to 85% is reported[24].

No previous literature analyzing the prevalence of osteochondrosis or spinal deformities in amputees could be found. We assume that patients experiencing those diagnoses also suffer from back pain.

To the author’s knowledge, there are no studies that found results in agreement with our study. This might be caused by only analyzing amputees with already diagnosed OA. In our amputees group, there is likely an number of patients with undiagnosed OA. Compared to the control group, this number might be increased, since an amputation strongly influences daily life and self-perception [25]. In some individuals this might reduce the perception of back pain or joint pain associated with the onset or mild OA.

In general, several other factors not registered in our database promote the development of OA and spinal disorders, including physical work and strenuous activities (e.g. skiing, tennis, soccer, golf, etc.) [26,27]. Those are likely to be underrepresented in the amputee group [13,28].

### Study limitations

The presented study has several limitations. First, separating patients in more active and less active while thresholding the costs for the treatment which is not accurate under all circumstances. It is easily imaginable that there are active patients with more cost-effective treatments that were not included in our group. Secondly, this study relies on data of one German health insurer that might influence the type of treatment. Third, a more detailed explanation of the results is prohibited by the lack of information on the duration of prosthetic treatment of the patients. Furthermore, the time of ascertainment of OA is unknown, thus there is a chance of OA occurring before the amputation. Fourth, matching of the groups could only be done based on age, gender and drug expense. Fifth, the severity of the OA is not coded in the ICD and therefore not included in the analysis. Kulkarni et al. reported an altered distribution of OA grades in amputees towards a more severe form (Kellgren-Lawrence Grade >3) compared to the non-amputee population (ratio of grade >3 to grade >2: amputees: 0.41 and 0.70 (amputated side vs non-amputated side); non-amputees: 0.22) [9]. However, in that study a very special subgroup of veterans with solely traumatic reasons for amputation was analyzed, which does not necessarily represent a typical amputee group. Other factors related to health status, like obesity, and play an important role in the development of OA, could not be considered since they were not available in the database. However, the authors regard those limitations as acceptable given the comparable high number of included cases. Despite our efforts to include only active and mobile patients with an amputation, it is unclear to which extent this was successful as such data is not considered in the examined database.

Since we expected to find increased prevalence of hip and knee OA in the amputee group as previous literature suggested due to increased loadings of joints, there must be one source of influence that was not accounted for. Literature suggests that amputees are considerably less active than non-amputees [13,28]. The authors found less development of OA despite increased load on amputees’ joints. This reduced activity therefore seems to overcompensate the increased joint loadings.

## Conclusion

Contrary to expectation and previous studies, we found a significantly decreased prevalence of OA and specific disorders of the spine in transfemoral amputees compared to a control group. The amputees with OA are significantly younger than patients with OA in the control group. The results of the study need to be used cautiously due to the major limitation of the study which is the lack of detail in individual patients caused by the methodology.

## Supporting information

S1 Table Age distribution of the includes patients and control group. Age distribution of patients diagnosed with the six selected diagnoses regarding musculoskeletal disorders based on the 10^th^ revision of the International Classification of Diseases (ICD)[18].
(XLSX)

S2 Table Age distribution of the population in Germany. Age distribution of the population in Germany and in the individual federal states. The data were collected within the framework of the large census in 2011 [17].
(XLSX)

S3 Table Mean age of the observed groups. Calculation of the mean age of the amputees and the five control groups with the observed diagnoses.
(XLSX)

S4 Table Raw data of amputees and control group from the insurer. Raw data of the insurer with the number of affected amputees and the control group over all ICD codes based on the 10^th^ revision of the International Classification of Diseases [18]. On the basis of these data, the statistical analysis of the first objective was carried out using the Chi-squared test.
(XLSX)
